# Hidden features: CD36/SR-B2, a master regulator of macrophage phenotype/function through metabolism

**DOI:** 10.3389/fimmu.2024.1468957

**Published:** 2024-12-17

**Authors:** Yuge Chen, Xuejia Zhang, Shengbin Huang, Maria Febbraio

**Affiliations:** ^1^ Mike Petryk School of Dentistry, Faculty of Medicine and Dentistry, College of Health Sciences, University of Alberta, Edmonton, AB, Canada; ^2^ School and Hospital of Stomatology, Wenzhou Medical University, Wenzhou, China; ^3^ Institute of Stomatology, School and Hospital of Stomatology, Wenzhou Medical University, Wenzhou, China; ^4^ Department of Prosthodontics, School and Hospital of Stomatology, Wenzhou Medical University, Wenzhou, China

**Keywords:** CD36, SR-B2, macrophage metabolism, tumor microenvironment, obesity, atherosclerosis

## Abstract

Once thought to be in a terminally differentiated state, macrophages are now understood to be highly pliable, attuned and receptive to environmental cues that control and align responses. In development of purpose, the centrality of metabolic pathways has emerged. Thus, macrophage inflammatory or reparative phenotypes are tightly linked to catabolic and anabolic metabolism, with further fine tuning of specific gene expression patterns in specific settings. Single-cell transcriptome analyses have revealed a breadth of macrophage signatures, with some new influencers driving phenotype. CD36/Scavenger Receptor B2 has established roles in immunity and lipid metabolism. Macrophage CD36 is a key functional player in metabolic expression profiles that determine phenotype. Emerging data show that alterations in the microenvironment can recast metabolic pathways and modulate macrophage function, with the potential to be leveraged for therapeutic means. This review covers recent data on phenotypic characterization of homeostatic, atherosclerotic, lipid-, tumor- and metastatic-associated macrophages, with the integral role of CD36 highlighted.

## 1 Introduction

CD36, also known as Scavenger Receptor B2, is a multiligand receptor found in most cells and tissues (1–3). In the myeloid lineage, CD36 is expressed by monocytes, macrophages, dendritic cells, megakaryocytes, microglia and platelets. Versatile in function, CD36 recognizes and binds danger- and pathogen-associated molecular patterns (DAMPs and PAMPs) as a result of cell infection or pathology, apoptotic cells, native and modified forms of lipoproteins, fatty acids, Thrombospondin-1, Histidine-Rich Glycoprotein, diacylglycerides, amyloid-β and azapeptides (4–19). Extensively studied, CD36 remains an enigma due to its chameleon-like quality to adapt its behavior to circumstances, and its ability to perform multiple functions within the same cell.

CD36 plays a key role in innate immunity against infectious diseases by recognizing infected host cells due to PAMPs and through recognition of alterations in the phospholipid bilayer (e.g. oxidation, nitrosylation, exposure of inner leaflet phospholipids, etc.) (20). CD36 in its capacity as a pattern recognition receptor contributes to macrophage phagocytic clearance of infectious pathogens (21). As a result, CD36 may influence the course of diseases caused by bacteria, virus, fungus and parasites. A few examples: In malaria, CD36 plays several roles, including endothelial cell-mediated parasite sequestration, macrophage phagocytic clearance and modulation of immune cell cytokine release. These actions do not result in parasite eradication, but may decrease host mortality by controlling parasitemia levels (22–25). CD36 protects against *Klebsiella* pneumoniae infection by enhancing lipopolysaccharide (LPS) responsiveness, boosting cytokine production and phagocytosis (20). CD36 might influence the uptake and processing of viral particles. Human immunodeficiency virus (HIV)-1 viral proteins, including negative factor (Nef) and p17, can affect the innate immune system by impairing the oxidative burst response and phagocytosis by monocytes/macrophages through regulation of CD36 (26, 27). CD36-specific antibodies block HIV-1 release from infected primary macrophages and thus viral transmission to T cells (28). HIV-1 infected patients exhibit increased lipid levels and monocyte activation, with greater CD36 and TLR4 expression on monocytes, indicating a key role in inflammation (29, 30). In patients with impaired T-cell immunity, macrophage CD36 has been shown to play a critical role in the recognition of disease-associated β-glucans associated with yeasts *Candida albicans* and *Cryptococcus neoformans*, which serve as necessary inducers of the innate immune response (31).

Not all actions of CD36 are protective, however. In *Mycobacterium tuberculosis* infection, CD36 induces M2 macrophage differentiation, enhances cytokine secretion, and suppresses macrophage migration and T cell activation, leading to inflammation and immune suppression (32). Additionally, in the lungs, alveolar macrophage CD36-mediated uptake of surfactant provides lipid for increased cellular demands as a result of mycobacteria infection, and supports parasite growth (33). In this review, we will focus on emerging roles of CD36 in macrophages that impact metabolism.

## 2 CD36 essentially contributes to metabolic pathways that define macrophage phenotypes in homeostasis and disease

### 2.1 Homeostatic macrophages

CD36’s effects on macrophages are manifold. As a receptor for lipoproteins, apoptotic cells, PAMPs and DAMPs, and a facilitator of fatty acid transport, CD36 plays an integral operative role, while also influencing differentiation and ultimate phenotype. Macrophages are functionally heterogeneous; they develop from bone marrow-derived monocytes or tissue-resident macrophages along a spectrum in response to situational factors and retain plasticity (34). They include those that prioritize pathogen killing, have immunosuppressive functions, or are involved in tissue homeostasis and wound healing. In the classic M1/M2 paradigm, CD36 is more highly expressed in macrophages associated with tissue homeostasis, remodeling and repair after injury (35–38). These homeostatic and tissue restoration macrophages store and oxidize fatty acids for the intermittent or extended energy needs involved in efferocytosis, phagocytosis of tissue debris and synthesis of degradative enzymes. Indeed, phagocytosis of cells couples with fatty acid oxidation by providing substrate that would otherwise contribute to excess lipid accumulation. Reparative macrophages show greater uptake of apoptotic cells than those involved in pathogen killing, and in this way decrease tissue exposure to necrosis and mitigate inflammatory responses (39). In a model of ischemia-induced brain injury, infiltrating blood monocyte-derived macrophages showed greater phagocytic activity, with concomitant increase in cell membrane CD36 (37). This was accompanied by elevated expression of lysosomal acid lipase, a critical mediator of the M2 phenotype through modulation of fatty acid supply for oxidation, through breakdown of triglycerides and lipoproteins (36). Inhibition of CD36 with antibody or drug significantly decreased phagocytosis, and altered the development of tissue-resolution macrophages as a result (37). In a more recent study, pathogenic polymorphisms in CD36 that reduced expression by ~50%, predisposed to myocarditis in patients immunized with the Pfizer-BioNTech BNT162b2 vaccine against COVID-19 (15). This was mechanistically linked to impaired efferocytosis. The authors noted that the effect was predominantly in M1 macrophages, but this is confounded by the fact that CD36 is necessary for expression of M2 markers (35, 36, 38). In both these cases, the functional role of CD36 was aligned with tissue repair and homeostasis; loss of CD36 function altered phenotype.

**Figure 1:**
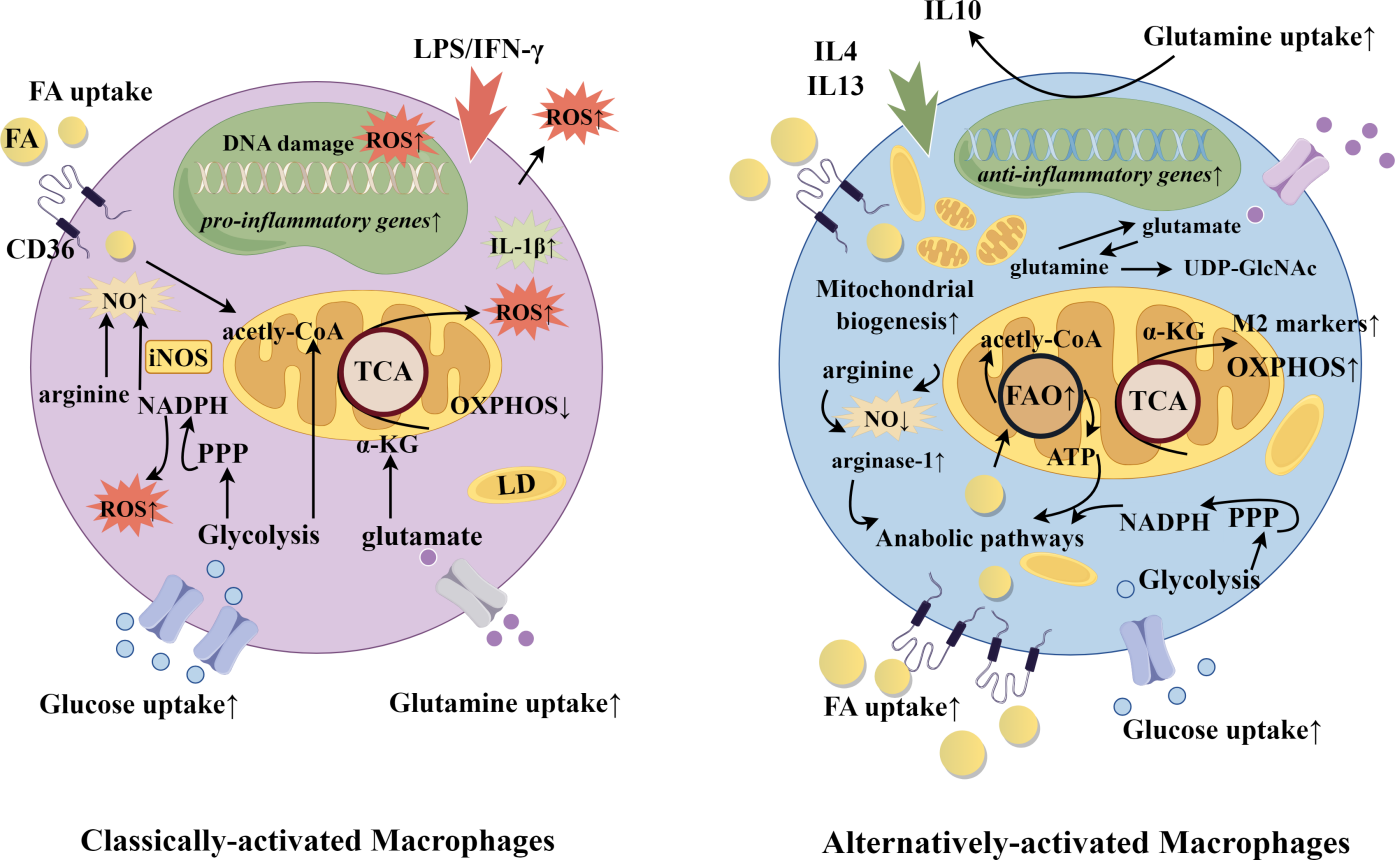
Classically-activated and alternatively-activated macrophages. Selected gene and metabolic pathways in classically-activated and alternatively-activated macrophages. Macrophage fate is induced according to exposure to diverse stimuli. Classically-activated macrophages are determined by IFNγ or lipopolysaccharide, while alternatively-activated macrophages receive signals from IL4 and IL13. Functionally, this yields upregulation of either more catabolic and inflammatory pathways or more anabolic and phagocytic pathways. In alignment, CD36 expression is greater in alternatively-activated macrophages to deliver fatty acids for oxidation and energy, and to provided substrate for the biosynthesis of membranes and other lipid-derived products. CD36 also plays a role in efferocytosis, through recognition of danger-associated and pathogen associated molecular patterns (DAMPs and PAMPs). FA, fatty acid; FAO, fatty acid oxidation; iNOS, inducible nitric oxide synthase; LD, lipid droplet; LPS, lipopolysaccharide; NADPH, nicotinamide adenine dinucleotide phosphate; NO, nitric oxide; oxPhos, oxidative phosphorylation; PPP, pentose phosphate pathway; ROS, reactive oxygen species; TCA, tricarboxylic acid cycle; UDP-GlcNAc, Uridine diphosphate N-acetylglucosamine. The figure was drawn by Figdraw (www.figdraw.com).

Reparative macrophages synthesize and secrete collagen, transforming growth factor-β, fibroblast growth factor, platelet-derived growth factor, and vascular endothelial growth factor (40–42). They are the conductors of the orchestrated remodeling process, attracting fibroblasts, stem cells, endothelial cells and others necessary in a temporal and coordinated manner. M2 macrophages also maintain a homeostatic environment through the synthesis and secretion of the anti-inflammatory cytokine IL10, and arginase-1, which converts arginine to ornithine and in this way thwarts the production of nitric oxide used in cytotoxic bursts (43). Classically activated (by interferon-γ or lipopolysaccharide) M1 and alternatively activated (by IL4 and IL13) M2 phenotypes demonstrate starkly different metabolic profiles: the overall ratio of oxidative phosphorylation to aerobic glycolysis has been shown to be 10-fold higher in M2 macrophages compared to M1 macrophages (36). Inhibition of fatty acid oxidation using the drug etomoxir decreased M2 signature gene expression, suggesting that the M2 phenotype is tightly associated with cellular metabolism (36). In line with this, delivery of triglycerides to the cell through CD36 was found to be necessary for M2 differentiation, and cells from mice and humans deficient in CD36 showed decreased expression of M2 markers (36). More recently, increased fatty acid oxidation as a necessary contributor to the immunosuppressive phenotype has been questioned (44). Differences in arginine and glutamine metabolism, and the channeling of fatty acids into anabolic pathways may be more essential in the determination of M1 *vs* M2 fate (44). Notwithstanding, uptake of lipids by CD36 remains a significant factor. A summary of metabolism-driving function for classical and alternatively activated macrophages is shown in **Figure 1**.

### 2.2 TREM2 and lipid-associated macrophages

Single-cell RNA sequencing has greatly enhanced characterization of macrophage subtypes, and revealed key influencers in their phenotype. Triggering receptor expressed on myeloid cells (TREM) 2 has emerged as an important driver of subgroups of macrophages in obesity, metabolic dysfunction-associated steatohepatitis (formerly non-alcoholic steatohepatitis) and atherosclerosis (45–47). Lipid-associated macrophages (LAMs), a distinct population that expand and accumulate in the stromal-vascular fraction from obese human visceral adipose tissue and adipose tissue from diet-induced obese mice, have a TREM2-directed transcriptional signature that includes CD36 and other genes responsible for lipid uptake and storage (46). Kyoto encyclopedia of genes and genomes pathway analysis revealed enrichment of those related to phagocytosis, endocytosis and lipid metabolism. Other pathways upregulated in LAMs were peroxisome proliferator-activated receptor (PPAR)- γ signaling and oxidative phosphorylation (46). Deficiency in TREM2 did not alter the expansion of the monocyte/macrophage compartment in adipose tissue, but resulted in loss of the associated biomarkers, indicative of its obligatory role in specialization of this population (46). In studies in TREM2-knockout (KO) mice, loss of this macrophage subset had profound ramifications: “massive” adipocyte hypertrophy, rapid weight gain, hypercholesterolemia, increased body fat and serum insulin, and insulin-resistance (46). These data suggest that in the obese setting, TREM2 LAMs attempt to maintain homeostasis through adipose tissue remodeling with an important role for CD36. Unlike homeostatic macrophages, LAMs occur in diseased states, including obesity, metabolic syndrome, fatty liver and cancer. They may differentiate from monocytes recruited to the tissue or from resident macrophages. Homeostatic resident macrophages are self-renewing. LAMs differ from homeostatic resident macrophages by expression of a gene program that includes TREM2, FABPs 4 and 5, lipase A, apolipoprotein E (apoE) and CD36, and a focus on lipid uptake and metabolism (48). Morphologically, LAMs are distinguished by the presence of lipid droplets.

In single-cell transcriptomic analyses of human atherosclerotic plaques, studies have consistently identified a distinct myeloid cluster containing “foamy macrophages” (49–51). These macrophages are characterized by abundant lipid, upregulation of fatty acid utilization pathways and a less inflammatory cytokine profile. Similar analyses done in mouse atherosclerotic lesions revealed a conserved subtype (51, 52). In both species, expression of TREM2 was a determining characteristic. Analogous to LAMs, these macrophages showed enrichment for lipid and fatty acid uptake and metabolic pathways, activation of liver X receptor/retinoid X receptor, which increase cellular lipid efflux mechanisms, and deceased cholesterol synthesis (45, 52). These cells were further characterized by expression of smooth muscle cell actin, but in the context of myeloid-determining transcription factors, and anti-inflammatory pathways driven by signal transducer and activator of transcription (STAT) 6, a downstream effector of IL4 (49). As was the case for TREM2 LAMs in the obese setting, CD36 was upregulated (45, 49–53). As a receptor for oxidized and native lipoproteins and fatty acids, it likely plays a major role in the development of the foamy macrophage morphology.

In both the setting of obesity and atherosclerosis, these TREM2 macrophage subpopulations function in uptake and disposal of excess lipid. They utilize various pathways: fatty acid oxidation to burn lipids for power, efflux mechanisms to remove excess, and finally storage routes. In sustained conditions, however, lipid overload leads to endoplasmic reticulum (ER) stress and autophagy (54). Cells deficient in TREM2 were more sensitive to ER stress; this suggests that the upregulation of TREM2 and associated genes in early lesions is compensatory and pro-survival (52, 53, 55). Additionally, macrophage deficiency of TREM2 led to a greater number of apoptotic cells in atherosclerotic lesions (52). In mouse atherosclerosis studies in which TREM2 was deficient in monocytes/macrophages, hematopoietic cells or globally, there was no consistent change in necrotic core size, however (45, 52, 53). The discrepancies may have to do with the timing of analysis, the cell types deficient in TREM2 and the model itself. Increased numbers of apoptotic cells and increased necrotic core size functionally track to a defect in efferocytosis. Although not addressed, this may be a consequence of CD36 downregulation in TREM2 KO cells, as uptake of apoptotic cells is a scavenger receptor function. This would suggest that as lesions progress, loss of TREM2 can have negative consequences. Relevant to this point, in a database analysis of carotid endarterectomy samples from recent stroke patients and non-stroke controls, TREM2 was enriched in the non-stroke patients, suggesting that TREM2 promoted a stable plaque phenotype (52).

**Figure 2:**
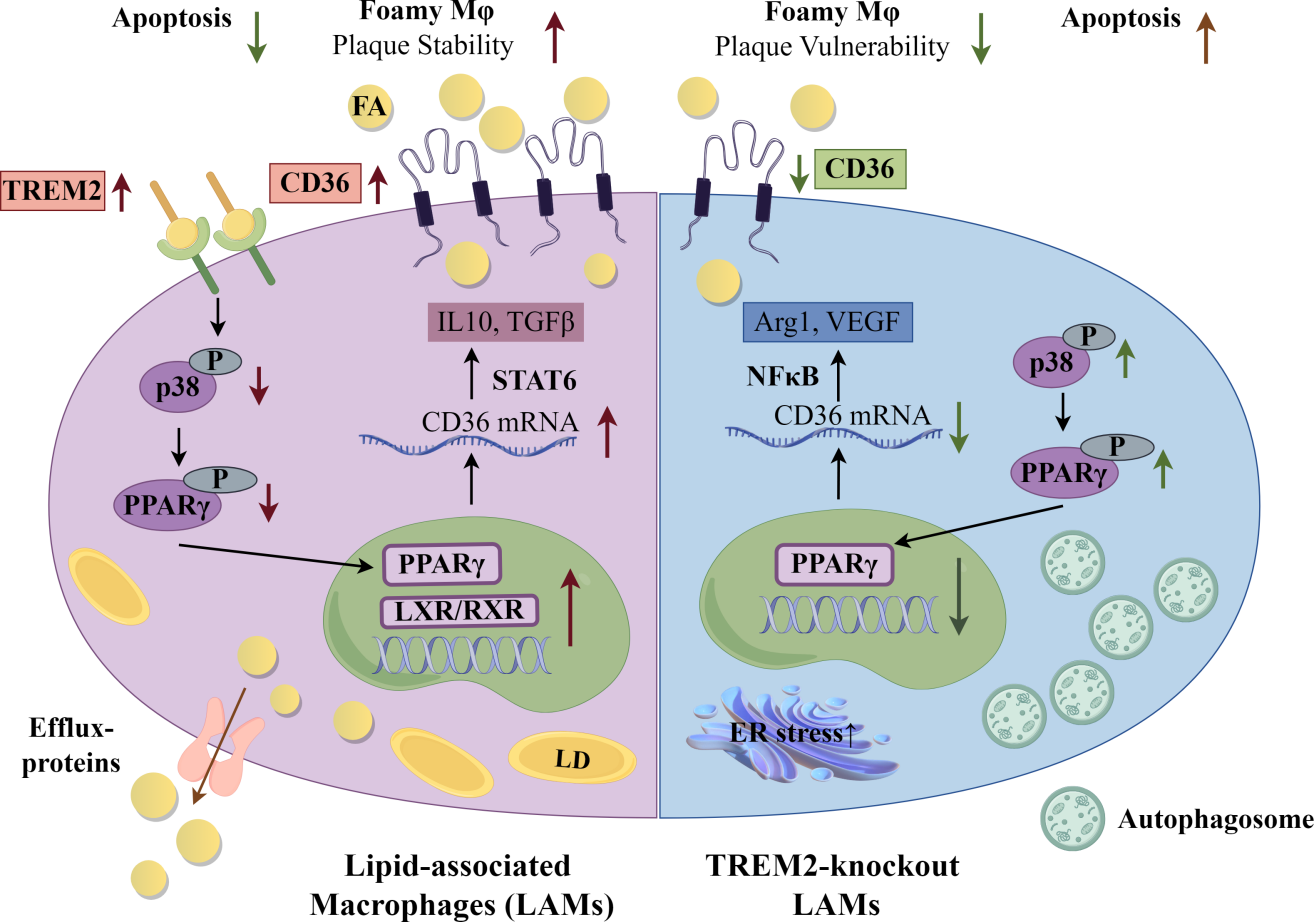
Lipid-Associated Macrophages (LAMs) and Triggering receptor expressed on myeloid cells (TREM) 2 KO Macrophages. Selected gene and metabolic pathways in LAMs and TREM2 KO macrophages. A foamy macrophage subset, labeled lipid-associated macrophages (LAMs), present in obesity and atherosclerosis have as a driver of gene signature Triggering receptor expressed on myeloid cells (TREM) 2. Upregulation of CD36 intensifies lipid influx. From a mechanistic perspective, TREM2 restrains the phosphorylation of p38 mitogen-activated protein kinase and peroxisome proliferator activated-receptor (PPAR) γ, consequently enhancing the nuclear transcriptional activity of PPARγ and subsequently facilitating the transcription of CD36. Arg1-arginase 1; FA, fatty acid; LD, lipid droplet; LXR, liver X receptor; NFκB, nuclear factor kappa B; RXR, retinoid X receptor; STAT6, signal transducer and activator of transcription 6; TGFβ, transforming growth factor β; VEGF, vascular endothelial growth factor. The figure was drawn by Figdraw (www.figdraw.com).

Interestingly, in these studies of global, hematopoietic and monocyte/macrophage-specific TREM2 KO mice, atherosclerosis *decreased* in both the apoE KO and low-density lipoprotein receptor (LDLR) KO, classic mouse models of atherosclerosis (45, 52, 53). TREM2 KO macrophages were shown to have decreased expression of CD36 *in vitro* and in plaques, and decreased oxidized LDL (oxLDL) uptake and lipid accumulation, respectively (45). In experimental models where TREM2 was targeted with agonistic antibodies, there was consensus that plaques showed greater signs of stability and reduced necrotic cores (53, 56). In one model, however, there was no change in overall lesion size, while in the other, overall lesion size was greater with increased number of macrophages, due to better survival and increased proliferation in plaques (53, 56). Complementary *in vitro* studies showed that antibody agonism led to increased oxLDL uptake, increased cholesterol efflux and cell survival (56). Thus, functionally, TREM2 induced higher expression of CD36 that then significantly impacted cellular metabolism. CD36-accrued lipid was channeled to be metabolized or effluxed as a protective macrophage survival mechanism, but the accumulation of foamy macrophages increased plaque size. Deficiency of TREM2 in macrophages led to smaller lesions, dependent upon loss of CD36 function, but paradoxically, the cells in those lesions were more vulnerable to death. A summary of the effects of TREM2 on metabolism and macrophage function and effects of deficiency are shown in **Figure 2**.

### 2.3 Caspase recruitment-domain containing protein 9, adenosine monophosphate activated protein kinase and atherosclerosis

The significant impact of CD36-dependent lipid uptake on macrophage phenotype is further illustrated in mice deficient for caspase recruitment-domain containing protein 9 (CARD9). This adapter protein is essential for activation of mitogen-activated protein kinases by Toll-like receptors (TLRs) (57, 58). OxLDL has been shown by multiple groups to be an endogenous ligand for TLRs; thus, CARD9 may play a role in amplification of pro-inflammatory signaling through TLR activation of p38 and Jun N-terminal kinase (59, 60). There have been several reports on the role of CARD9 in atherosclerosis with conflicting results (61, 62). Recent mechanistic investigation, using multiple mouse models (apoE KO, LDLR KO, recombination activation gene (RAG) 2 KO, CD36 KO, CARD9 KO and combinations) to rule out effects of the adaptive immune system and gut microbiota, has shown that CARD9 deficiency increased atherosclerosis lesion burden, with marked enhancement in macrophage and necrotic core lesion areas (63). These mice had a less pro-inflammatory cytokine profile and no significant differences in serum cholesterol levels (63). Further investigation showed that cells deficient in CARD9 had greater oxLDL uptake and lipid accumulation, and oxLDL-mediated apoptosis (63). CD36 cell membrane expression was greatly increased and shown to be essential to the increase in atherosclerosis, macrophage and necrotic core areas *in vivo* (63). Mechanistically, upregulation of CD36 not only contributed to increased macrophage foam cell formation, but also resulted in lipid overload that impaired autophagy pathways, leading to enhanced and progressed lesion development (63). Autophagy was impaired as a result of inhibition of adenosine monophosphate-activated protein kinase (AMPK) and downstream activation of mammalian target of rapamycin (mTOR) C1/mTORC2 (63).

Other studies have also revealed CD36-mediated lipid uptake as an essential regulator of AMPK activation. AMPK is a cellular sensor of nutritional status, and through phosphorylation of key effector proteins, directly or indirectly regulates metabolism with far-reaching consequences (64). As a general statement, AMPK activation leads to an increase in catabolic pathways and an inhibition of anabolic pathways (64, 65). AMPK is phosphorylated as a result of an increase in the AMP to adenosine triphosphate (ATP) ratio (64, 65). AMPK increases the activities of glycolysis, fatty acid oxidation and autophagy, and induces mitochondrial biogenesis, while inhibiting lipid, protein, glucose, glycogen, sterol and ribosomal RNA (rRNA) synthesis, to bring the cell back to homeostasis (64, 65). In order to increase cellular levels of ATP, AMPK enhances fatty acid uptake, to couple with catabolic pathways (66–68). Hence, AMPK activation results in increased CD36 expression and localization to the plasma membrane.

While an association between TREM2 and AMPK in macrophages was not noted in studies of TREM2 KO mice, TREM2, SIRT1 and PPAR-γ, all necessary for shifts in energy metabolism in macrophages, have been shown to be tightly linked to AMPK and required for anti-inflammatory polarization of microglia, the resident myeloid cells of the brain (69). In microglia, TREM2 upregulates CD36 to promote phagocytosis of amyloid-β (70).

**Figure 3:**
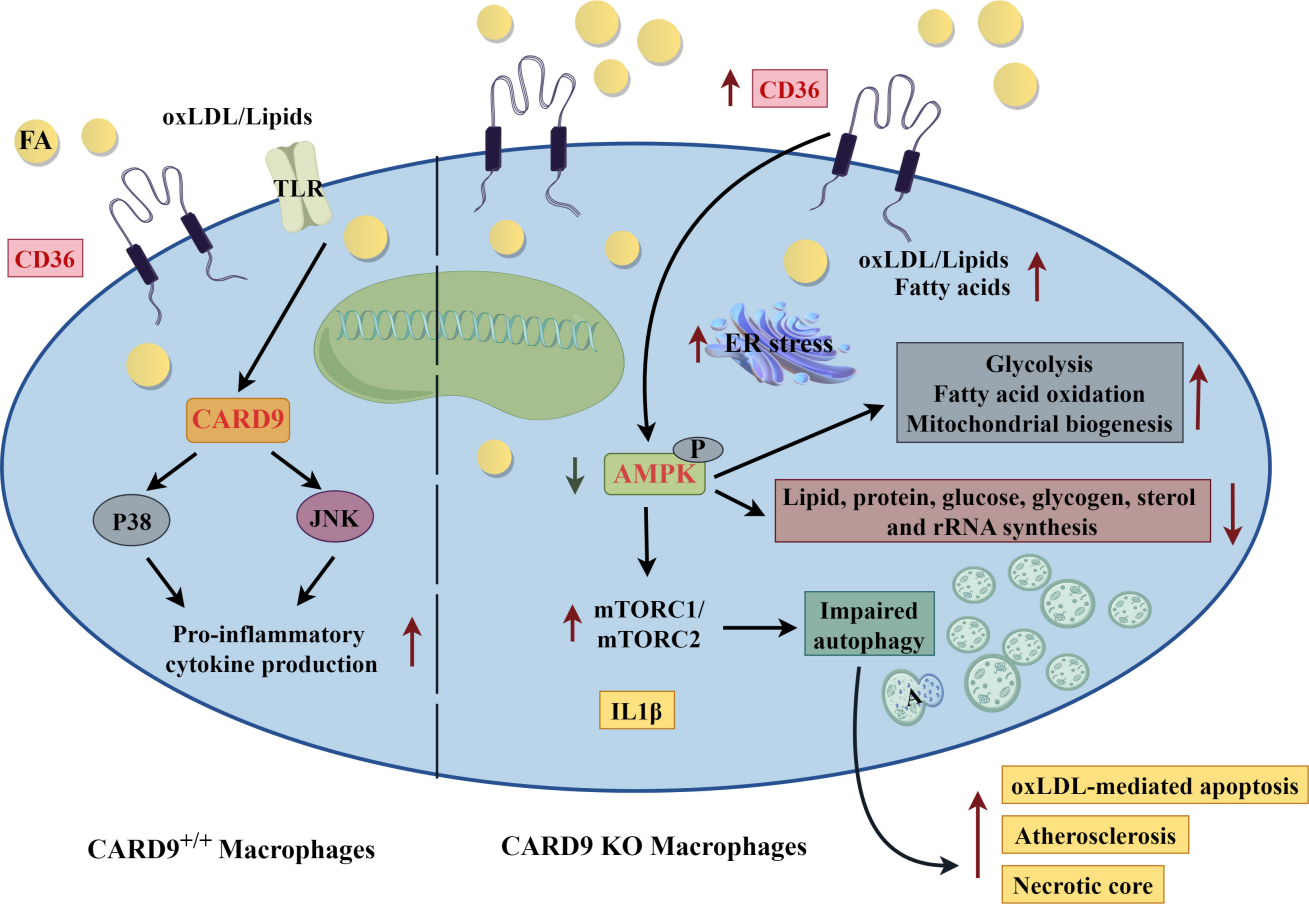
Caspase Recruitment-Domain Containing Protein 9 (CARD9), adenosine monophosphate activated protein kinase (AMPK) and Atherosclerosis. Mechanism of increased atherosclerosis in CARD9 deficient macrophages. In atherosclerosis, oxidized low-density lipoprotein (oxLDL) is a ligand of Toll-like receptor (TLR). CARD9 is an adapter protein that links TLR to p38 and jun N-terminal kinase (jnk) to amplify pro-inflammatory signaling pathways (left side of diagram). Card9 deficiency (right side of diagram) increases CD36 expression leading to greater uptake of lipid and foam cell formation. Lipid overload subsequently results in endoplasmic reticulum (ER) stress. Adenosine monophosphate activated protein kinase (AMPK) phosphorylation is decreased and mammalian target of rapamycin (mTOR) activation is increased in CARD9 KO macrophages, leading to impaired autophagy, increased apoptosis and necrotic core, and overall, accelerated atherosclerosis. A, autophagosome; FA, fatty acid. The figure was drawn by Figdraw (www.figdraw.com).

Mechanistically, AMPK-mediated increased expression of CD36 has been studied in cells other than myeloid cells. In intestinal cells, increased CD36 expression by AMPK was due to inhibition of polyubiquitination by Parkin (68). In myocytes, CD36 was found in a complex with the Src kinase, Fyn and liver kinase B1 (LKB1) (67). Increased levels of circulating fatty acids led to displacement of Fyn from the complex, leading to LKB1 phosphorylation of AMPK, and downstream effects (67). Interestingly, in myocytes, CD36 not only regulated AMPK activation, but then was itself regulated by AMPK. Stimulated recruitment of CD36 to the membrane by insulin, contraction or AMPK was dependent upon AK strain transforming (AKT)substrate 160 and Rab-GTPase to control vesicular trafficking and increased plasma membrane localization (66). Whether similar mechanisms account for AMPK-induced CD36 increased expression and localization in myeloid cells remains unknown. The mechanism of increased atherosclerosis in CARD9-deficient macrophages is shown in **Figure 3**.

### 2.4 Tumor-associated macrophages

A major therapeutic initiative in cancer is targeting lipid metabolism as a way to reprogram immune cells in the tumor microenvironment (TME) (71, 72). This niche is unique due to an often immature and disorganized vasculature that limits supplies of oxygen and nutrients, and the removal of debris and waste products, while supporting tumor cell proliferation and immune cell emigration (73). Given the highly proliferative capacity of tumor cells, there is a significant increase in anabolic pathways, including lipid, cholesterol, rRNA and protein (74). Tumor cells develop strategies for sustained cellular biosynthesis via aerobic glycolysis (the Warburg effect) and glutamine-dependent anaplerosis, which provides substrates for a modified tricarboxylic acid cycle (74–76). While inefficient in generating ATP, it has been estimated that lactate production from aerobic glycolysis occurs at a faster rate (10-100x) compared to mitochondrial oxidation; thus, the net amount of ATP produced would be comparable (77). Additionally, increased glucose uptake and breakdown are hypothesized to feed into necessary biosynthetic pathways by providing carbon for the synthesis of proteins, lipids and nucleotides, nicotinamide adenine dinucleotide phosphate equivalents necessary for *de novo* fatty acid synthesis, and regeneration of nicotinamide adenine dinucleotide to keep glycolysis going (75, 76).

Evidence shows that high-fat diets and obesity contribute to more than a dozen cancers (78, 79). Tumor-driven modifications of adipose tissue enhance tumorigenesis of the breast, ovary, prostate, lung and bone marrow, amongst others (80–83). While fatty acid oxidation is disfavored in tumor cells, fatty acids can be utilized and stored for the biosynthesis of membranes and lipid signaling molecules (72). Thus, dietary and tumor cell-synthesized fatty acids are used to produce arachidonic acid, docosahexaenoic acid and eicosapentaenoic acid, eicosanoids, prostaglandins and lysophosphatidic acid (72). These can act autonomously or on other cells in the TME to influence phenotype. Prostaglandin E2 (PGE2), the product of the enzyme cyclooxygenase 2, which is preferentially expressed in cancer cells (e.g., colon, prostate, melanoma, breast and lung), has emerged as an important player in influencing TME cellular immunosuppressive phenotypes (84, 85).

Tumor-associated macrophages (TAMs) are found abundantly in solid tumors and evidence suggests that they play a significant role in modulation of other immune cells in the microenvironment through the expression of cytokines, growth factors and other products (44). As is true of macrophages in general, the phenotypes of TAMs exist along a spectrum, with pro- and anti-tumorigenic properties (86). Tumor-derived mediators and products activate signaling pathways that define both the metabolism and behavior of TAMs (86). Although numerous and with the capacity to mount an anti-tumor immune response, TAMs are often pro-tumorigenic.

Isolated TAMs from various human and mouse cancers have been shown to be lipid-rich and to functionally have enhanced lipid uptake via CD36 (87). Metabolism of fatty acids is an essential determinant of TAM phenotype (87, 88). Interfering with fatty acid oxidation, for example, using the drug etomoxir, blocked the development of immunosuppressive TAMs (87, 88). Concomitant with increased suppressive phenotypes was increased expression of CD36 (87). Significantly, etomoxir-treated TAMs or those isolated from CD36 KO tumors did not have a suppressive phenotype and blocked the proliferation of multiple tumor cell types in *in vitro* co-culture experiments (87).

In the analysis of human tumors, single-cell sequencing data from breast and lung cancers revealed that CD36 is predominantly expressed on TAMs, and that these TAMs have significantly higher expression of CD36 compared with control tissues (87). Elevated expression of CD36 in TAMs correlated with genes involved in TAM differentiation and fatty acid oxidation (87). Published gene array datasets from renal cancer patients again showed that CD36 expression correlated with a TAM gene expression signature (87). In gene array datasets from glioblastoma patients, there was a strong correlation between the genes involved in fatty acid oxidation and CD36 (87). This supports work in mice as relevant to human cancers, and that metabolic programming of TAMs is CD36-dependent. Taken together, these data provide support for immunomodulation through metabolic manipulation as a potential therapeutic approach in cancer.

### 2.5 Metastasis-associated macrophages

The liver is a major site of tumor cell metastasis and patients with liver metastases have an overall poor prognosis. Similar to TAMs, metastasis-associated macrophages (MAMs) have been phenotypically characterized in mice and humans and the data compellingly suggest an essential role for CD36 in an immunosuppressive, pro-tumorigenic role (89, 90). Metastatic Lewis lung carcinoma cells injected intraperitoneally into the spleen resulted in fewer metastatic nodules, overall decreased tumor area, reduced tumor proliferation and blood vessel number in CD36 KO mice compared to controls. Contribution from hepatocyte CD36 was ruled out using liver-specific CD36 KO mice (90). Depletion of macrophages with clodronate liposomes suppressed liver metastasis; liver metastases were also markedly reduced in macrophage-specific CD36 KO mice (90).

MAMs isolated from tumors had an increased number of lipid droplets compared to macrophages isolated from non-diseased liver (90). MAMs had higher expression of CD36 and increased uptake of fatty acids. Notably, the expression of other fatty acid uptake proteins (Fatty Acid Binding Proteins 1-6) was unchanged or decreased (90). In this model, the investigators uncovered a novel delivery mechanism for fatty acids derived from tumor cells: extracellular vesicles (90). These vesicles were shown to transfer fatty acids to MAMs, and they were subsequently trafficked to the mitochondria. Uptake of these tumor-derived vesicles correlated with CD36 expression and was significantly decreased in CD36 KO MAMs (90).

Like TAMs, MAMs were predominantly immunosuppressive. This suggests that the TME in some way promotes such a phenotype. Analysis of lipids in the liver metastatic TME showed enrichment for unsaturated and monounsaturated fatty acids; *in vitro* experiments demonstrated that CD36 expression was required for the immunosuppressive phenotype mediated by these lipids (90). In particular, oleic acid was shown to have an anti-inflammatory effect on bone marrow-derived myeloid cells and promoted the development of immunosuppressive dendritic cells and macrophages (88, 91). The mechanism was linked to metabolism: upregulation of pathways related to mitochondrial respiration, fatty acid oxidation, lipid droplet formation and downregulation of fatty acid synthesis pathways and desaturases. Oleate decreased the expression of mature macrophage markers, increased expression of signature TAM markers, including those associated with an inhibitory phenotype, and induced high levels of arginase activity (88, 91). Remarkably, these experiments revealed that oleate alone was sufficient to induce the differentiation of TAMs at phenotypical and functional levels. *In vivo*, short-term feeding of mice a diet supplemented with oleic acid increased macrophages with a suppressive phenotype in mesenteric adipose tissue (92).

Lipid droplets, which are a prominent common characteristic of immunosuppressive macrophages, play a key role in maintenance of phenotype: they provide a reservoir of fatty acids to support the metabolism and functions of the immunosuppressive state (88). Interference with enzymes and associated proteins involved in mobilization of fatty acids from lipid droplets disrupted the development of the immunosuppressive phenotype (88, 91, 93).

**Figure 4:**
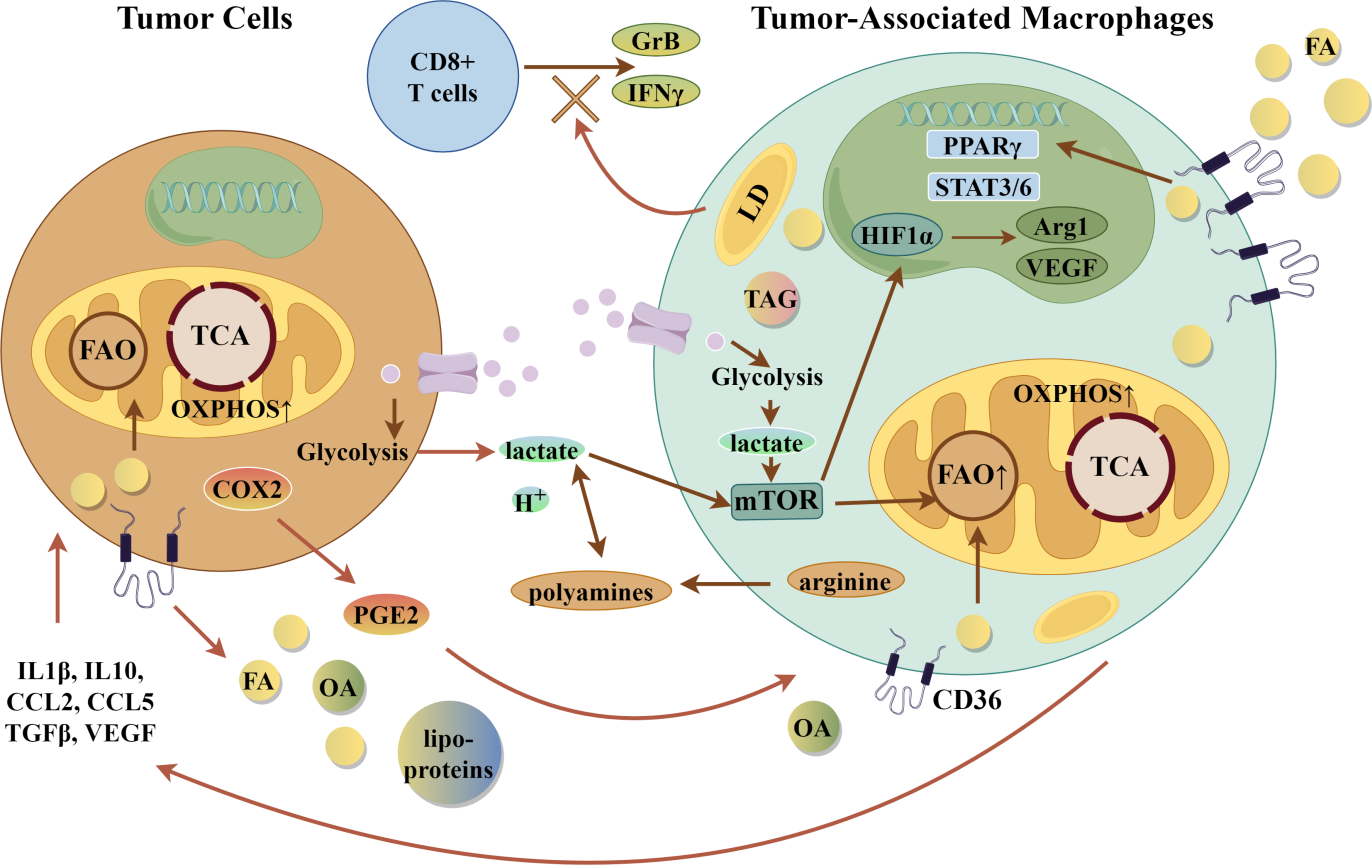
Tumor-Associated Macrophages (TAMs). Integration of Metabolic Pathways with suppressive phenotype in TAMs. Selected gene and metabolic pathways in TAMs that lead to suppressive effects on tumor cells. Arg1, arginase 1; CCL2/5, CC-chemokine ligand 2 or 5; COX2-cyclooxygenase 2; FA, fatty acid; FAO, fatty acid oxidation; GrB, granzyme B; H^+^- acid; HIF1α, hypoxia-inducible factor 1α; LD, lipid droplet; mTOR, mammalian target of rapamycin; OA, oleic acid; oxPhos-oxidative phosphorylation; PGE2, prostaglandin E2; PPARγ, peroxisome proliferator activated-receptor γ; STAT3/6, signal transducer and activator of transcription 3 and 6; TAG, triacylglyceride; TCA, tricarboxylic acid cycle; TGFβ, transforming growth factor β; VEGF, vascular endothelial growth factor. The figure was drawn by Figdraw (www.figdraw.com).

When the immune cell population of the TME was examined in macrophage-specific CD36 KO mice compared with controls, there was a diminished number of MAMs and an increased number of B cells and T cells, including cytotoxic CD8+ T cells (90). Expression of interferon-γand granzyme B were also significantly increased in these cytotoxic CD8+ T cells, suggesting greater anti-tumor potential. In human hepatic metastases, CD36 expression correlated with an M2-like, immunosuppressive phenotype in two datasets examined (90). These studies bolster the hypothesis that CD36 expression is essential to support the TAM and MAM immunosuppressive phenotype by contributing to lipid metabolism. **Figure 4** summarizes key metabolic and suppressor pathways in TAMs.

The mechanism of CD36 upregulation in TAMs and MAMs is likely multifactorial. IL4 is a differentiating cytokine for M2 macrophages. Due to the oxidative stress in the TME, macrophages are exposed to IL4 in the context of oxLDL, and this has been shown to have a strong influence on CD36 expression (94). IL13, important in the differentiation of TAMs, also upregulates CD36 (95). Secretion of long-chain fatty acids, especially saturated and monounsaturated classes, likely leads to a feed-forward response that increases CD36 expression (90). Similarly, as the macrophages increase fatty acid metabolism and oxidation, this also has a feed-forward effect, driving up the expression of fatty acid uptake receptors, including CD36. Some of these same molecules also activate PPARγ, which again increases CD36 expression (96).

### 2.6 Myeloid-derived suppressor cells

In addition to TAMs, there are also myeloid-derived suppressor cells (MDSCs) in the TME (97–99). MDSCs are divided into polymorphonuclear (PMN-MDSCs) and monocytic (MO-MDSCs) subtypes (97, 99). They are recruited to the TME as a result of signals similar to TAMs. PMN-MDSCs stimulate T-cell tolerance in an antigen-specific manner, while MO-MDSCs are potently immunosuppressive and exert effects in an antigen-independent manner (97, 100). MDSCs are believed to arise in response to sustained pressure on myelopoiesis as a result of chronic infections, cancer and autoimmune diseases (100). The initial proliferation and training of MDSCs occurs in the bone marrow and spleen, but importantly, the phenotype is then refined in the peripheral sites of disease, where condition-specific growth factors, cytokines and derived products exert influence (101).

**Figure 5:**
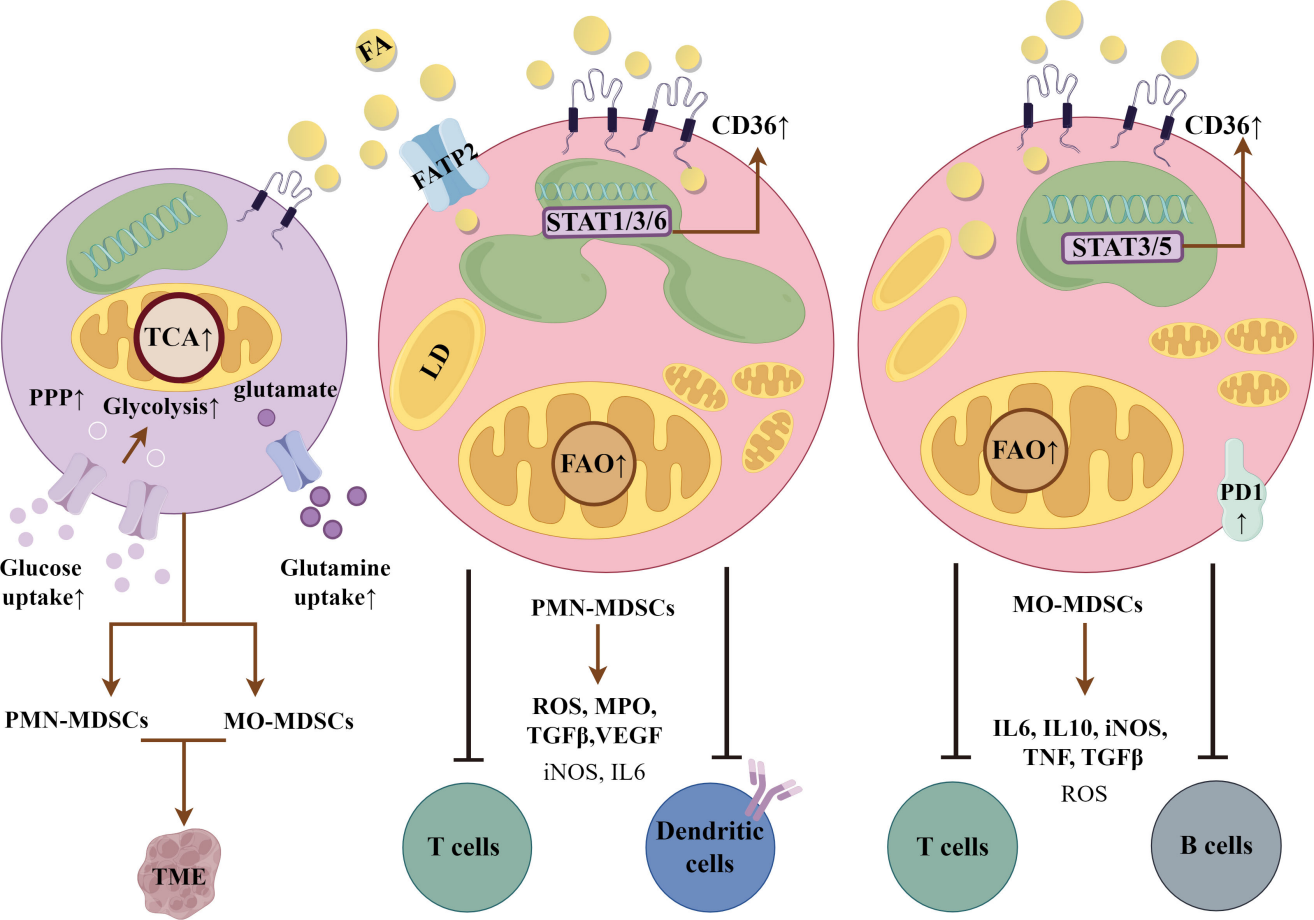
Myeloid-derived suppressor cells (MDSCs). Polymorphonuclear (PMN)-MDSCs and monocytic (MO)-MDSCs are derived from a common bone marrow precursor (1^st^ cell on left). They are then educated in the tumor microenvironment (TME), dependent upon the factors and products expressed. Both are dependent upon fatty acid oxidation (FAO) as a primary energy resource. CD36 expression supports the suppressive phenotype through uptake and delivery of fatty acids (FA), which are stored in lipid droplets (LD). PMN-MDSCs express reactive oxygen species (ROS), myeloid peroxidase (MPO), transforming growth factor β (TGFβ) and vascular endothelial growth factor (VEGF), and other genes downstream of signal transducer and activator of transcription 1, 3 and 6. They are immunosuppressive in an antigen-dependent manner. They upregulate fatty acid transport protein 2 (FATP2) to supplement FA uptake by CD36. MO-MDSCs have greater lipid droplet accumulation via CD36 and potent, non-antigen dependent immunosuppressive activity. They produce IL6, inducible nitric oxide synthase (iNOS), transforming growth factor β (TGFβ), tumor necrosis factor and programmed cell death protein-1 (PD-1), which interacts with programmed cell death-ligand 1 on immune cells to exert immunosuppressive control. CD36 expression is induced in a STAT3/5 dependent manner. The figure was drawn by Figdraw (www.figdraw.com).

During differentiation, reports show that MDSCs have high rates of glycolysis, glutamine uptake and reliance on the tricarboxylic acid cycle and pentose phosphate pathway for energy (102). Investigations have shown that activation of MDSCs in the TME led to upregulation of CD36 via STAT3 and STAT5, and increased lipid uptake (103). Phenotyping of spleen *vs.* tumor-infiltrating MDSCs revealed significant differences in mitochondrial mass, oxygen consumption and fatty acid oxidation, all increased in those cells educated in the TME (97). As has been suggested for other immunosuppressive myeloid cells, fatty acid oxidation was the source of intermediates for the biosynthesis of fatty acids, amino acids, proteins and ribose for rRNA. These feed into biosynthetic pathways for anti-inflammatory molecules, including IL10, programmed cell death protein-1 and inducible nitric oxide in MO-MDSCs. The development of PMN-MDSCs has also been shown to be dependent upon lipid metabolism to produce reactive oxygen species and myeloid peroxidase, which limit dendritic cell antigen presentation and T-cell activation (104). Tumor-secreted PGE2 and fatty acid transport protein (FATP) 2 play essential roles in their determination (85, 105). Thus, strategies that interfere with FATP2 or CD36 curb MDSC suppressive effects on effector cells (105, 106). While many of these studies have been done in mouse models, recent single-cell RNA sequencing analyses showed greater than two-fold increases in CD36 expression in MDSCs from head and neck and breast cancer (107). A comparison between PMN-MDSCs and MO-MDSCs is shown in **Figure 5**.

## 3 Mechanisms of CD36 expression and function in macrophage phenotypes

The PPARγ pathway is one of the primary regulators of CD36, particularly in the context of lipid metabolism and anti-inflammatory responses in macrophages (108, 109). PPARγ is a member of the nuclear hormone receptor superfamily and is activated by various ligands, including fatty acids. Upon activation, PPARγ forms a heterodimer with retinoid X receptor (RXR), which binds to PPAR response elements on the CD36 gene promoter, enhancing its transcription. This pathway is particularly active in tissue-reparative M2 macrophages, where it promotes lipid uptake, fatty acid incorporation into membranes and organelles, storage, and anti-inflammatory functions (96). Fatty acids are inducers of CD36 mRNA and protein levels, consistent with this (110, 111). Dysregulation of PPARγ and subsequent alterations in CD36 expression are implicated in various metabolic disorders.

In the hyperlipidemic environment of LAMs, free fatty acids and cytokines, as a result of underlying chronic inflammation, are the likely drivers of CD36 expression in adipose tissue. The chemokine CCL2 recruits monocytes into inflammatory sites, where they differentiate into macrophages in response to the cytokines macrophage colony-stimulating factor and IL4; the latter have also been shown to increase CD36 expression (112–115). This is also dependent on the expression and activation of PPARγ and 12/15 lipoxygenase, which generates 13-hydroxyoctadecadienoic acid (HODE) and 15-hydroxyeicosatetraenoic acid, transcriptional activators of PPARγ (116).

While the pathway underlying TREM2-dependent CD36 expression in LAMs was not defined, in microglia, in response to the CD36 ligand, amyloid β, TREM2 induced CD36 expression via phosphoinositide-3 kinase (PI3K)/AKT signaling, resulting in upregulation of CCAAT-enhancer-binding protein (C/EBP) α, a transcriptional activator of CD36 (70). Mutation of the C/EBPα binding site in the promoter of CD36 suppressed CD36 upregulation. C/EBPα is necessary in the development of both pro-inflammatory M1 and homeostatic M2 macrophages, suggesting a master role in macrophage polarization (117). This also suggests that there is other input to define the final macrophage phenotype.

In atherosclerosis, in addition to fatty acids and cytokines, oxidized lipids, such as oxLDL, and cholesterol crystals drive CD36 expression. Previous work established that cholesterol crystals trigger nucleotide-binding oligomerization domain, leucine-rich repeat and pyrin domain containing (NLRP) 3 inflammasome formation, as well as nicotinamide adenine dinucleotide phosphate (NADPH) oxidase and xanthine oxidase-dependent ROS production (118, 119). This, in turn, activates Bruton’s tyrosine kinase, which phosphorylates p300, which next activates STAT1 acetylation and binding to the STAT binding site in the CD36 promoter to enhance its activity. Site-directed mutagenesis of the CD36 promoter STAT binding site abolished increased CD36 expression mediated by cholesterol crystals (119).

Induction of CD36 expression by oxLDL, like fatty acids, has been shown to be due to the activation of PPARγ (108, 109). OxLDL and its components linoleic acid, 9-HODE and 13-HODE, were established as potent activators of PPARγ activity (108). Atherogenic macrophage expression of CD36 is thus perpetuated by a cycle in which oxLDL drives its own uptake through internalization of PPARγ ligands and activators, 9-HODE and 13-HODE, which upregulate expression of CD36. Significantly, TREM2 has been shown to also impact the PPARγ pathway, and this may underlie the TREM2-CD36-dependent transition to foamy macrophages. TREM2 was shown to inhibit p38 phosphorylation, resulting in reduced downstream phosphorylation of cytoplasmic PPARγ (45). As a result, there was increased transit of PPARγ to the nucleus which increased the transcription of CD36, promoting cholesterol crystal, oxLDL and fatty acid uptake.

The liver X receptor (LXR) and RXR heterodimer is another critical regulator of CD36 in macrophages, with significant implications for cholesterol metabolism, inflammatory responses and atherosclerosis (120). Upon activation by oxysterols found in oxLDL, LXR/RXR upregulates CD36 and other genes involved in lipid handling, enhancing cholesterol efflux and reducing intracellular lipid accumulation. Impaired LXR/RXR signaling contributes to foam cell formation and plaque progression. The LXR/RXR pathway’s influence on CD36 expression underscores its role in balancing pro- and anti-inflammatory responses in macrophages. The importance of this pathway in the regulation of CD36 and other cholesterol-handling genes was recently demonstrated. Myeloid LXR deficiency was shown to dramatically increase atherosclerosis, with increased numbers of TREM2 foamy macrophages (121). Single-cell RNA sequencing uncovered that the lack of LXR altered the expression of expected TREM2 target genes: there was a marked switch to a pro-inflammatory phenotype, with reduction of cholesterol-handling and efflux proteins, promoting increased atherosclerosis (121). These studies show the intersection of TREM2 with already established pathways of CD36 expression.

Not only does oxLDL drive an increase CD36 expression, it also contributes to the reprogramming of macrophages in atherosclerosis. In a recent report, oxLDL was shown to stimulate increased intracellular ROS in a CD36-dependent manner, resulting in a change in macrophage phenotype as a result of ROS-dependent nuclear factor-kappa B (NFκB) activation (122). Mechanistically, CD36 altered macrophage metabolism by shifting fatty acids away from oxidation and instead into the mitochondria, where they accumulated, leading to ROS generation (122). The resulting macrophage resembled a pro-inflammatory, pro-atherosclerotic M1 subtype.

While there is utility in characterizing macrophage phenotypes, it is also important to understand that in different scenarios, genes such as CD36 may be more or less expressed but retain significant roles. M1 macrophages may have lower levels of CD36 expression than M2, but still have differentiating effects. The nuclear factor kappa B (NFκB) pathway, a master regulator of inflammation, in general, has been found to play an opposing role in the regulation of CD36 (115, 123, 124). NFκB is typically activated in M1 macrophages, characterized by a pro-inflammatory phenotype. Activation of NFκB by pro-inflammatory stimuli such as LPS, tumor necrosis factor α, and IL1β leads to the translocation of NFκB subunits (p65 and p50) to the nucleus, where they promote the transcription of pro-inflammatory genes while inhibiting CD36 expression (125). This suppression aligns with the inflammatory role of M1 macrophages, which prioritize pathogen defense and tissue destruction over lipid handling. This pathway is also relevant in tumor microenvironments, where M1 macrophages are involved in tumor suppression and immune activation (126–128). Conversely, the STAT6 pathway, activated by IL4 and IL13, promotes M2 macrophage polarization and upregulates CD36, enhancing lipid uptake and supporting tissue repair and anti-inflammatory functions (129–131). This pathway is particularly relevant in fibrotic diseases, such as pulmonary fibrosis, where CD36-mediated lipid metabolism drives macrophages toward a reparative but pro-fibrotic phenotype.

The complexity of the tumor microenvironment presents a challenge to isolate specific signals that upregulate CD36 expression and stimulate immunosuppressive properties. A multitude of signaling pathways, including Janus kinase (JAK)/STAT, toll-like receptor and NFkB, mitogen-activated protein kinase, as well as those downstream of cytokines, chemokines, growth factors, tumor-secreted products and metabolites exist. It is much more probable that multiple pathways regulate the function of CD36 in these circumstances.

A distinctive trait of many solid tumors is hypoxia. Multiple reports have shown that hypoxia increases CD36 expression. In macrophages, upregulation of CD36 was dependent upon hypoxia-inducible factor (HIF) 1α and p38 (132). Sequencing of the CD36 promoter confirmed a HIF1 binding site (133). Lactic acid production and an acidic milieu are other characteristics of solid tumor environments. In hepatocellular carcinoma, CD36 expression in tumor cells was positively associated with increased glycolysis and lactic acid production (134). In this report, CD36 was observed to induce glycolysis through mTOR and the activation of the Src/PI3K/AKT signaling axis. Whether this is true of macrophage CD36 in TAMs and MAMs requires further study.

Oxidative stress is a hallmark of a number of diseases, including cancer and stroke. The ischemic brain after experimental injury shows an influx of monocyte-derived macrophages, and interestingly, compared with the uninjured hemisphere, these cells upregulate CD36 expression (37). Stroke-induced increased CD36 mRNA levels correlated with increased expression of lysosomal acid lipase, an M2 macrophage marker. Analogously, oxidative stress in the TME may upregulate macrophage CD36. HIF1-dependent upregulation of CD36 also resulted in increased uptake of oxLDL, which is likely present as a result of extensive ROS found in the TME (135). This then could trigger the feed-forward loop outlined previously, involving PPARγ and its lipid ligands.

The pathways responsible for CD36-dependent suppressive effects of macrophages in the TME have not been elucidated; however, there has been extensive study of the role of CD36 in the suppression of T cell anti-tumor responses (136). The presence of cholesterol in the TME was shown to increase CD36 expression by CD8+ tumor-infiltrating lymphocytes in human cancer, and this led to increased lipid uptake, accumulation, and peroxidation (137, 138). Cholesterol and lactic acid, as well as oxidized phospholipids, were shown to lead to CD36-dependent CD8+ tumor-infiltrating lymphocyte dysfunction (137–139).

Mechanistically, increased CD36 expression in human and murine CD8+ tumor-infiltrating lymphocytes correlated with decreased cytokine production and impaired antitumor activity. The latter was associated with increased lipid peroxidation, intracellular iron and ROS levels, and ferroptosis (136). Ferroptosis, an iron-dependent mechanism of cell death that targets mitochondria, results from a decrease in the activity of glutathione peroxidase 4, which also suppresses the metabolism of arachidonic acid, contributing to phospholipid peroxidation (140). Polyunsaturated fatty acids, which are abundant in the TME, reduce cytokine production and enhance ferroptosis, and this is dependent on the expression of CD36 (136). What remains unknown is whether these same pathways lead to CD36-dependent suppressive functions in other cell types.

## 4 Concluding remarks and future directions

While the transcriptome defines gene expression and cellular phenotype to a large extent, it has become more increasingly apparent that macrophage subtypes develop their functionality as a result of environmental cues and moreover, retain the ability to metamorphose. A predominant determining and sustaining factor in phenotypic outcome is cellular metabolism, which is essentially entwined with functional gene and biomarker expression. The microenvironment, oxygen-rich or poor, characterized by apoptotic or necrotic cells and tissue, and encompassing other cells, secreted products, bioactive mediators and waste, contribute to the spectrum of macrophage populations. Moving forward, the challenge will be to harness this knowledge to specifically reprogram macrophages in disease settings to overcome suppressive and loss-of-function states. CD36, an essential element in fatty acid and lipid uptake and metabolism, and in functions aligned with reparative, homeostatic and suppressive macrophage phenotypes, may be an attractive target.
